# Retraction: A Toxin-Antitoxin Module of *Salmonella* Promotes Virulence in Mice

**DOI:** 10.1371/journal.ppat.1009031

**Published:** 2020-10-21

**Authors:** The PLOS Pathogens Editors

Following the publication of this article [1], concerns were raised regarding the data presented in Fig 3D and S4 Fig.

Specifically,

- In Fig 3D, a straight vertical signal can be observed directly on the left of the band in lane 3, which appears to be inconsistent with the overall background of the blot. The corresponding author was unable to explain the presence of this signal.
- In S4 Fig, the bands and background signal of lanes 1, 2, and 7 appear similar. In addition, the background signal directly surrounding all bands presented in this panel appears to be inconsistent with the overall background of S4 Fig. The corresponding author has indicated that the image was not intended for publication.

The underlying data for the results presented in Fig 3D and S4 Fig are no longer available. In light of the concerns affecting multiple figure panels that question the integrity of these data, the *PLOS Pathogens* Editors retract this article.

JPG and SM did not agree with the retraction. MADlC agreed with the retraction. WZ, CF, GG, DR and CC either did not respond directly or could not be reached.
